# Five methodological considerations for validating LLMs in risk of bias assessment

**DOI:** 10.1017/rsm.2026.10073

**Published:** 2026-02-05

**Authors:** Vihaan Sahu

**Affiliations:** Georgian National University SEU, GE

To the Editors,

I read with interest the recent article by Eisele-Metzger and colleagues,^1^ which provides a timely investigation into the use of the large language model (LLM) Claude 2 for assessing the risk of bias (RoB) in randomized controlled trials. The exploration of LLMs to address the substantial resource burden of systematic reviews is of great importance.^2^ However, to ensure this line of inquiry yields robust and actionable insights, several methodological considerations warrant deeper discussion. I wish to raise five key critiques grounded in the broader literature on RoB reliability and LLM evaluation, which suggest opportunities to strengthen future validation studies and better define the practical role of LLMs in evidence synthesis.

## 1 Methodological concerns with practical implications: The imperfect reference standard

The study’s use of existing Cochrane RoB assessments as the reference standard is pragmatic but problematic, as human assessments themselves show low reliability. Minozzi et al.^3^ reported an overall inter-rater reliability (IRR) kappa of just 0.16 for RoB 2 among experienced reviewers, with domain-specific values ranging from slight (κ = 0.04) to moderate (κ = 0.45). Similarly, Armijo-Olivo et al.^4^ found very poor agreement (κ = 0.02) between Cochrane and external reviewer panels. Consequently, when Eisele-Metzger et al. report a Cohen’s κ of 0.22 between Claude and the Cochrane reference, it is unclear whether this reflects poor model performance or inherent noise in the “gold standard.”^3^ ^,^ ^4^ What proportion of disagreements stemmed from human inconsistency versus genuine model error? For review teams, this is not an academic concern; reconciling disagreements is a major time sink.^3^ ^,^ ^5^ A more valid approach would compare the LLM against a consensus from a panel of experts using a prespecified, piloted protocol, as demonstrated in more rigorous LLM evaluations.^6^ While the authors acknowledge the limitations of their reference standard in their discussion, this limitation could have been more thoroughly addressed in the study design through the use of a consensus-based reference standard.

## 2 Missed opportunity: LLMs as augmentation rather than replacement

While the authors rightly conclude that Claude cannot currently replace human assessors and appropriately recommend further investigation into “other models of support than providing stand-alone RoB judgements,”^1^ a more immediate and practical application may be as an augmentation tool within a hybrid workflow. Gartlehner et al.^7^ demonstrated that an LLM used for data extraction achieved 96.3% accuracy compared to humans, concluding that “integration of LLMs… should for now, only be done in the form of semi-automation.” This aligns with evidence of the immense resource demands of reviews, which take a mean of 67 weeks and 5 team members to complete.^8^ Building on the authors’ opening for alternative support models, I encourage exploring Claude’s output as a first-pass assessment, flagging trials or domains for focused human expert scrutiny. Could the authors conduct a secondary analysis simulating this workflow? How much human time could be saved if the LLM’s judgments were used as one of two independent assessments, with humans resolving discrepancies?^7^ ^,^ ^8^

## 3 Information asymmetry and its impact on valid conclusions

The evaluation may have systematically disadvantaged the LLM due to residual information asymmetry. Although Claude was provided with compressed protocols or register entries, this process and the exclusion of supplementary materials may not equate to the comprehensive information synthesis performed by Cochrane reviewers, who routinely consult a wider array of sources in their original, uncompressed form.^9^ This violates the principle of information parity crucial for fair comparison. Cierco Jimenez et al.^10^ note that ML tool performance is heavily dependent on the information they can access, while the PRISMA statement emphasizes comprehensive information gathering as foundational for a valid review.^11^ The qualitative differences in information access may have underestimated Claude’s true capability. Did the authors audit which disagreements were attributable to information limitations? Future evaluations must ensure LLMs and human raters operate on identical information sets to draw valid conclusions about comparative performance.^9^ ^,^ ^11^

## 4 Inadequate exploration of performance discrepancies across studies and domains

The authors note variability in Claude’s performance across studies but do not deeply investigate the causes. Heterogeneity in human–model agreement is well documented. Lai et al.^6^ find LLM performance varied significantly by RoB domain, and Hartling et al.^12^ show that agreement between human reviewers is influenced by trial characteristics (e.g., intervention type, funding source). Eisele-Metzger et al. report a domain-level kappa of 0.31 for “missing outcome data” but only 0.10 for “selection of the reported result.”^1^ What explains this threefold difference? Was performance poorer for industry-funded trials or subjective outcomes, as seen in human reliability studies?^12^ A qualitative analysis of cases with high versus low agreement could reveal whether errors cluster around specific methodological complexities or reporting ambiguities, guiding targeted improvements in prompt engineering or model training.

## 5 Temporal validity and the rapid evolution of LLMs

The study evaluates a specific version of Claude 2, but LLM capabilities are evolving at an unprecedented pace. Singhal et al.^13^ document rapid improvements in clinical knowledge encoding, while Chen et al.^14^ demonstrate that GPT-3.5 and GPT-4 exhibited significant behavioral drift, sometimes improving, sometimes degrading over just a few months. This poses a major challenge for evaluation reproducibility.^14^ Weidinger et al.^15^ argue for frameworks for ongoing evaluation of AI systems. The findings of Eisele-Metzger et al. are thus a snapshot of a moving target. How can the field develop evaluation frameworks that remain relevant? I propose that validation studies incorporate mechanisms for continuous re-evaluation, report model version and date explicitly, and use benchmarks that can track progress or regression over time.^14^ ^,^ ^15^

## 6 Conclusion

The work by Eisele-Metzger et al.^1^ is a valuable step in exploring AI for evidence synthesis. However, to translate this promise into reliable practice, the methodology must mature. Fundamental issues include the use of noisy human reference standards, a narrow focus on replacement over augmentation, information asymmetry in evaluations, and inadequate scrutiny of performance heterogeneity. Most critically, the rapid evolution of LLMs threatens the temporal validity of single-point assessments.

I propose a coordinated approach to LLM evaluation for RoB assessment with five components: (1) standardized benchmarks using expert consensus reference standards^3^ ^,^ ^6^; (2) evaluation frameworks that assess augmentation potential and workflow integration, not just replacement capability^7^ ^,^ ^8^; (3) protocols ensuring strict information parity between human and AI assessments^9^ ^–^ ^11^; (4) detailed, qualitative analyses of factors influencing performance heterogeneity across domains and trial types^6^ ^–^ ^12^; and (5) mechanisms for continuous evaluation to track performance as models evolve.^13^ ^–^ ^15^

The ultimate goal in evidence synthesis must be to leverage technology to produce high-quality, timely evidence for healthcare decisions. By addressing these methodological considerations, we can ensure that the integration of LLMs into systematic review methodology is both rigorous and transformative.

## Data Availability

None declared.
